# Self-Tuning of Signal Detection Level for Energy Detection-Based Carrier Sense in Low-Power Wide-Area Networks

**DOI:** 10.3390/s24113368

**Published:** 2024-05-24

**Authors:** Shusuke Narieda, Takeo Fujii

**Affiliations:** 1 Graduate School of Engineering, Mie University, Tsu 514-8507, Mie, Japan; 2 Advanced Wireless and Communication Research Center (AWCC), The University of Electro-Communications, Chofu 182-8585, Tokyo, Japan; fujii@awcc.uec.ac.jp

**Keywords:** carrier sense, energy detection, low-power wide-area networks

## Abstract

Carrier sense allows end devices to improve the communication quality through autonomous decentralization by consuming power. In particular, energy detection-based carrier sense can improve communication quality compared with peak detection-based carrier sense. To improve the trade-off between communication quality and energy consumption in low-power wide-area networks (LPWANs), this study proposes a self-tuning method for the signal detection level of an energy detection-based carrier sense, that is, the carrier sense level in sub-GHz band LPWANs. In the proposed method, the carrier sense level of each end device is determined based on the reception success probability of the acknowledgment packet, such that they become low carrier sense levels for an end device with low probability and high carrier sense levels for an end device with high probability. The proposed method enables autonomous decentralized derivation of the carrier sense level using only existing protocols. Numerical examples show that the proposed method can improve the performance of end devices with a high path loss to a gateway.

## 1. Introduction

The Internet of Things (IoT) is a structure that enables information interchange between an extremely large number of various things with physical sensors via wireless networks. Several pieces of information can be extracted by connecting things to the network, and they can be analyzed on the Internet cloud and shared, regardless of location. Through this process, the value of information obtained from various sources increases, and this may provide a solution to various social problems. Several systems that use this structure, such as industrial applications [1,2] and smart cities [3], have been developed and investigated.

The low-power wide-area network (LPWAN) [4] is one of the candidates for wireless infrastructures of such IoT systems owing to their characteristics. LPWA communications are characterized as energy-efficient wireless and long-range communications, although the data rate of the communication is very low. Furthermore, employing LPWANs does not inhibit the rapid spread of IoT systems because a private or local network can be constructed using LPWAN in unlicensed bands; for example, 920 MHz bands are utilized for LPWA communications in Japan. The characteristics of LPWANs are suitable for IoT systems with an extremely large number of battery-driven end devices. However, the frequency band may be under strain, leading to the degradation of the communication quality in the frequency band.

Carrier sense [5,6], which is employed in IEEE 802.11 [7] based on wireless local area networks, is a powerful technology that enables the improvement in the communication quality even in an environment in which a significant number of wireless transceivers are transmitting and receiving their packets. It is well known that carrier sense technologies attempt to detect communication signals from other end devices in the channel as transmitted packets. If signals from other end devices are not detected, the end device transmits packets immediately; if the signals are detected, after waiting for a while, the system checks again with the carrier sense whether the channel is idle. Carrier sense utilizes a signal detection technology [8] to detect the communication signals, such as peak detection, energy detection [9], or cyclostationary detection [10]. Peak detection is the simplest signal detection technique that determines the presence or absence of the target signals whether the peak power of the received signals within a signal detection period exceeds a threshold value. For example, the carrier sense level in the Japanese 920 MHz band is defined as the peak power of received signals to avoid interference to co-existing passive tag systems, and peak detection can be detected at the carrier sense level. In ARIB STD-T108 [11], which is a regulation for 920 MHz LPWANs in Japan, packet transmission from the end device is strictly limited by several wireless parameters, namely the transmission power, duty-cycle and transmission time for one packet. However, the purpose of these methods is solely to protect co-existing systems, and the peak detection-based carrier sense cannot improve the performance of the end devices well due to the low accuracy of signal detection owing to the noise floor, especially in LPWANs, where the sensitivity is lower than that of the noise floor.

There is very little research on carrier sense in LPWANs, which have various wireless parameters, such as communication protocols, bandwidth, noise floor levels, and sensitivity, that are completely different from wireless LANs. This is because only a few countries require carrier sense for LPWA communication, and one of them is Japan. In this situation, to overcome this problem and improve communication quality, carrier sense technologies based on energy detection for LPWANs have been investigated [12]. Energy detection is a signal detection technique that determines the presence or absence of target signals whether the average power of the received signals within the signal detection period exceeds a threshold value. The accuracy of energy detection is superior to that of peak detection. When energy detection is applied to carrier sense, the target signals do not occupy the carrier sense period due to the short packets in LPWANs, and the characteristics differ from those of the general signal detection problem, that is, under the assumption that the length of the target signal is infinite. In [12], several characteristics of these cases are investigated both theoretically and numerically. However, in the energy detection-based carrier sense, the signal detection level of the carrier sense, that is, carrier sense level, depends on the carrier sense period, which determines the energy consumption for carrier sense at the end device. Therefore, it is important to discuss the trade-off between the accuracy of the carrier sense and energy efficiency [13,14].

In this study, we discuss a simple method for determining the signal detection level of the energy detection-based carrier sense, including whether the carrier sense is executed or not. To consider the trade-off between the accuracy of the carrier sense and energy efficiency, the optimization of the carrier sense level can be considered under the constraint of the energy consumed in the entire network. However, the optimization problem for the entire network is unrealistic because new protocols are required to distribute the optimization results computed at the gateway. Therefore, autonomous decentralized determination is expected at each end device. We propose a self-tuning method of the carrier sense level for the energy detection-based carrier sense in the Sub-GHz band LPWANs.

The remainder of this paper is organized as follows. Section 2 presents related works of the proposed self-tuning method and the original contribution of this paper. In Section 3, we define LPWAN system models and present the energy detection-based carrier sense. Section 4 presents the proposed self-tuning method for autonomously determining the signal detection level of the energy detection-based carrier sense. Section 5 presents the numerical analysis results of LPWAN with the energy detection-based carrier sense. Finally, Section 6 presents the conclusions of this study.

## 2. Related Works and Original Contribution

### 2.1. Related Works

Autonomous decentralized processing methods for the LPWA end devices have been previously proposed [15,16,17,18]. An efficient mechanism for autonomous slot assignment based on time-slotted long-range wide-area networks (LoRaWANs) has been proposed [15,16]. In [17], an autonomous adaptive frame size protocol for LoRa has been proposed. Furthermore, medium access control protocol to determine transmission parameters for the end devices based on their location to the gateway has been proposed [18]. On the other hand, for example, LoRaWAN has an adaptive data rate (ADR) [19], which controls the data rate of the end devices according to the communication environment. ADR enables the autonomous decentralized control at the end devices in LoRaWAN, and several methods of performance improvement based on ADR have been proposed [20,21,22].

All of these related show that some specialized communication protocols are required. Therefore, these protocols cannot directly adopt another LPWA communication scheme, such as wireless smart utility networks (Wi-SUNs). The proposed method can adopt several LPWA communications as long as these have the acknowledgment mechanism.

### 2.2. Original Contribution

The main contribution of this paper can be summarized as follows:

1. We propose a simple self-tuning method for the carrier sense level for the energy detection-based carrier sense in the Sub-GHz band of the LPWAN. The proposed method can autonomously determine the carrier sense level of each end device.
2. Unlike these works shown in the previous subsection, we employ acknowledgment (ACK) protocols, which are already implemented in most LPWA communications, in LPWANs for the proposed self-tuning method. For example, all end devices for LoRaWANs must implement Class A, which specifies ACK packets [19], while Wi-SUN equips the ACK frame [23]. The signal detection level of the energy detection-based carrier sense determines whether the reception of ACK packets is successful or not. By employing ACK protocols, the autonomous decentralized determinations of the carrier sense level can be realized for each end device. Therefore, the proposed method is applicable to all LPWA schemes with the ACK mechanism, and this is an advantage of the proposed method.
3. Numerical examples show that the proposed method can solve the trade-off between the accuracy of the carrier sense and energy efficiency under the given target packet delivery ratio.

## 3. Preliminary Notion

### 3.1. LPWAN

In this study, a simple LPWAN model composed of geographically fixed *K* end devices and one gateway located at the center of the communication area with a radius *R* m is investigated. The LPWAN is a fundamentally pure ALOHA-based wireless network with the energy detection-based carrier sense. However, ACK packets are transmitted and received, as necessary. All end devices transmit packets conveyed to each observed data point every $T_{TX}$ s after the carrier sense to confirm the channel usage of other end devices. The end device executes the carrier sense $N_{SC}$ times at random intervals and does not transmit the packet if it cannot confirm that the channel is idle. We employ a simple radio propagation model in which the received signal power $P_{RX,k}$ from the *k*th end device at the gateway is determined only by the transmission power $P_{TX,k}$ as follows:

(1) $$\begin{array}{c} P_{RX,k}=P_{TX,k}-L_{k},\;k=1,\cdots ,K, \end{array}$$

where $L_{k}$ is the path loss between the gateway and *k*th end device in decibels, which decays exponentially with distance as

(2) $$\begin{array}{c} L_{k}=-10\log_{10}\left(d_{k}^{\alpha_{GE}}f_{c}^{2}\times 10^{-2.8}\right),\;k=1,\cdots ,K, \end{array}$$

where $d_{k}$ m, $\alpha_{GE}$, and $f_{c}$ MHz are the distances between the gateway and *k*th end device, path loss exponent between the gateway and *k*th end device, and carrier frequency, respectively. Furthermore, we employ a path loss model $\Theta_{k,i}$ between the *k*th and *i*th end devices, as follows:

(3) $$\begin{array}{c} \Theta_{k,i}=-10\log_{10}\left(\bar{d_{k,i}^{\alpha_{GE}}}f_{c}^{2}\times 10^{-2.8}\right),\;k\neq i,\;k,i=1,\cdots ,K, \end{array}$$

where $\bar{d_{k,i}}$ and $\alpha_{GE}$ are the distance between the *k*th and *i*th end devices and the path loss exponent between the *k*th and *i*th end devices, respectively.

We divide the operation of the end devices into three parts: packet transmission, carrier sense, and sleep. We assume that the operation of the end device is sleep except for packet transmission and carrier sense. Based on these operations, the current consumption of the end device is presented to evaluate the performance of the proposed method. Table 1 lists the current consumption of the end devices for each operation. These values listed in Table 1 are obtained by using the LoRa calculator [24]. In Table 1, we assume that the current consumption of the carrier sense is almost the same as that of packet reception, as described in [25].

### 3.2. Energy Detection-Based Carrier Sense in LPWANs

In this subsection, the energy detection-based carrier sense in LPWANs is presented. We let $T_{P}$ and $T_{CS}$ denote the length of the packet and the carrier sense period, respectively. Note that this will occur not only for $T_{P}\leq T_{CS}$, but also for $T_{P}>T_{CS}$ because the presence of the interference packet is unknown during the carrier sense period. Considering these factors, the carrier sense success probability $P_{CS}$ of the energy detection-based carrier sense can be written as [12]

(4) $$\begin{array}{c} P_{CS}=\frac{1}{T_{P}+T_{CS}}\left[2\sum_{n=1}^{\min\left(T_{P},T_{CS}\right)}P_{D,A}\left(n\right)+\left(|T_{P}-T_{CS}|+1\right)P_{D,A}\left\{\min\left(T_{P},T_{CS}\right)\right\}\right], \end{array}$$

where $\min\left(X,Y\right)$ is a function that yields the minimum values of *X* and *Y*. In addition, $P_{D,A}\left(n\right)$ is the signal detection probability for a finite length target signal *n*. The signal detection probability for a finite packet length $T_{P}$ is given by

(5) $$\begin{array}{c} P_{D,A}\left(T_{P}\right)=Q\left\{\left(\frac{P_{CS}^{\left(mW\right)}}{\sigma_{v}^{2}+\min\left(1,\frac{T_{P}}{T_{CS}}\right)\sigma_{w}^{2}}-1\right)\sqrt{2T_{CS}BW}\right\}, \end{array}$$

where $\sigma_{v}^{2}$, $\sigma_{w}^{2}$, and $P_{CS}^{\left(mW\right)}$ are the variances in noise at the end device, arriving interference, and the threshold for signal detection in milliwatts, respectively. Furthermore, $Q\left(z\right)=\frac{1}{\sqrt{2}}\int_{z}^{\infty }e^{-t^{2}/2}dt$ [26].

**Figure 1:**
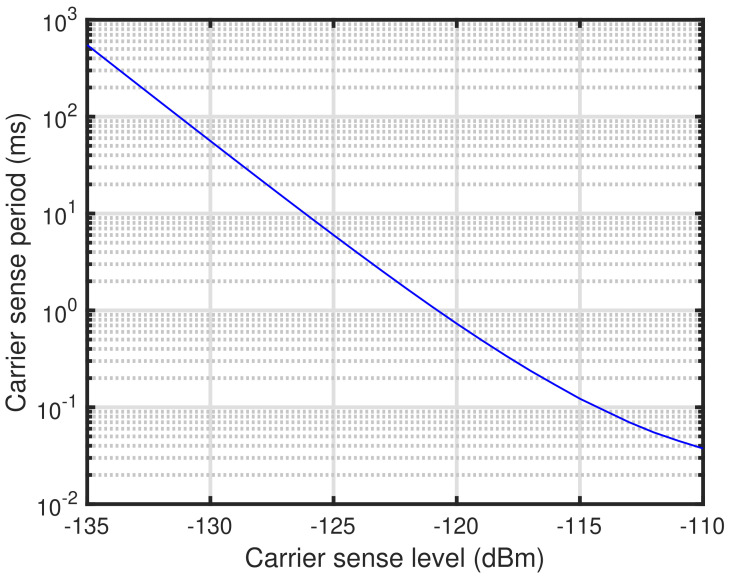
Relationship between carrier sense level and carrier sense period.

Inaccurate noise power estimation degrades the accuracy of the energy detection-based carrier sense due to the signal power to noise ratio (SNR) wall phenomenon [27]. To avoid this, a sufficiently long carrier sense period is required [28]. The carrier sense period $T_{CS}$ can also be written, resulting in the derivation of the signal detection theorem [12], as follows:

(6) $$\begin{array}{c} T_{CS}=\frac{1}{2\gamma^{2}BW}{\left\{Q^{-1}\left(\bar{P_{FA}}\right)-\left(1+\gamma \right)Q^{-1}\left(\bar{P_{D}}\right)\right\}}^{2} \end{array}$$

where $\gamma =\sigma_{w}^{2}/\sigma_{v}^{2}$, $\bar{P_{D}}$, and $\bar{P_{FA}}$ are the SNR, target signal detection probability, and target false alarm probability, respectively. Note that γ can also be interpreted as the SNR computed from the carrier sense level as a signal power and the noise floor. Figure 1 shows the relationship between the carrier sense level and the carrier sense period. In Figure 1, the channel bandwidth, noise figure, target signal detection probability, and target false alarm probability are 200 kHz, 6 dB, 0.99, and 0.01, respectively. As shown in Figure 1, it can be seen that the carrier sense period increases as the carrier sense level decreases. Due to the fact that the current consumption of the carrier sense can be assumed to be that of packet reception [25], this implies that a lower carrier sense level leads to higher current consumption. If the carrier sense level can be appropriately determined at each end device, neither too high nor too low, it is possible to reduce the extra current consumption at the end device. To achieve this, we propose the self-tuning method of carrier sense level for the energy detection-based carrier sense in the next section.

## 4. Self-Tuning Method of Signal Detection Level for Energy Detection-Based Carrier Sense

### 4.1. Overview of Proposed Method

The proposed self-tuning method enables the autonomous decentralization determination of the carrier sense level at each end device. To achieve this, the proposed method determines whether the ACK packet transmission is successful. A significant number of LPWA communication schemes equip the protocols of corresponding ACK packets.

The proposed self-tuning method can exploit the existing protocols; therefore, it is not necessary to develop new protocols for optimization. In the proposed self-tuning method, first, each end device is autonomously decentralized and determines the carrier sense level based on the ACK packet delivery ratio during a certain period. The period for the determination of the carrier sense level is called the self-tuning period with $L_{P}$ length. After this self-tuning period, ACK packets are used if necessary, according to the original purpose of the network. In this study, an application without ACK packets is assumed, that is, ACK packets are not used after the self-tuning period, to focus on the proposed method. In the next subsection, the self-tuning method for the determination of the carrier sense level is presented.

### 4.2. Self-Tuning Method for Determination of Carrier Sense Level

We assume that each end device has memory for maintaining the ACK reception results with $L_{M}$ length, and the *n*th result at the *k*th end device is stored as $r_{k}\left(n\right)$ with a value of 1 or 0. One $r_{k}\left(n\right)$ is used for the packet conveying the same data; that is, $r_{k}\left(j\right)$ will be 0 even if the packet cannot be transmitted due to carrier sense. After generating $L_{M}$ packets, the estimated *n*th packet delivery ratio at the *k*th end device ${\hat{P}}_{PDR,k}\left(n\right)$, which can be sequentially computed using the results stored in memory, can be written as

(7) $$\begin{array}{c} {\hat{P}}_{PDR,k}\left(n\right)=\frac{\sum_{j=n-L_{M}+1}^{n}r_{k}\left(j\right)}{L_{M}},\;k=1,\cdots K. \end{array}$$

The proposed self-tuning method enables the autonomous decentralized determination of the carrier sense at the end devices by sequentially computing Equation (7). We let $\bar{P_{PDR}}$ denote target packet delivery ratio. In the proposed self-tuning method, the carrier sense level decreases if ${\hat{P}}_{PDR,k}\left(n\right)<\bar{P_{PDR}}$, whereas it increases if ${\hat{P}}_{PDR,k}\left(n\right)\geq \bar{P_{PDR}}$. Furthermore, to reduce the current consumption at the end device, the end device does not execute the carrier sense if ${\hat{P}}_{PDR,k}\left(n\right)$ > $\bar{P_{PDR}}$ and the carrier sense level exceeds the upper bound of the variable range of the carrier sense level.

Thus, the carrier sense level at the *k*th end device $P_{CS,k}^{\left(dBm\right)}\left(n\right)$ depends on ${\hat{P}}_{PDR,k}\left(n\right)$ and $\bar{P_{PDR}}$. The decision-making process of the carrier sense level can be divided into two cases, that is, ${\hat{P}}_{PDR,k}\left(n\right)\geq \bar{P_{PDR}}$ and ${\hat{P}}_{PDR,k}\left(n\right)<\bar{P_{PDR}}$, as shown below.

#### 4.2.1. Case: ${\hat{P}}_{PDR,k}\left(n\right)\geq \bar{P_{PDR}}$

In this case, which represents rich communication quality at the *k*th end device, the carrier sense level is raised or the end device does not execute the carrier sense to reduce energy consumption. These processes can be written as

(8) $$\begin{array}{c} P_{CS,k}^{\left(dBm\right)}\left(n\right)=\left\{\begin{array}{cc} P_{CS,k}^{\left(dBm\right)}\left(n-1\right)+X_{dB}, & P_{CS,k}^{\left(dBm\right)}\left(n-1\right)<P_{CS,UB}^{\left(dBm\right)}\\ \infty , & P_{CS,k}^{\left(dBm\right)}\left(n-1\right)\geq P_{CS,UB}^{\left(dBm\right)} \end{array}\right.,\;k=1,\cdots K \end{array}$$

where $X_{dB}$ and $P_{CS,UB}^{\left(dBm\right)}$ are the step sizes of the carrier sense level in decibels and the upper bound of the variable range of the carrier sense level in the proposed method, respectively. Note that $P_{CS,k}^{\left(dBm\right)}\left(n\right)=\infty$ indicates that the end device does not execute the carrier sense.

#### 4.2.2. Case: ${\hat{P}}_{PDR,k}\left(n\right)<\bar{P_{PDR}}$

In this case, which represents poor communication quality at the *k*th end device, the carrier sense level is lowered or the end device executes the carrier sense. These can be written as

(9) $$\begin{array}{c} P_{CS,k}^{\left(dBm\right)}\left(n\right)=\left\{\begin{array}{cc} P_{CS,k}^{\left(dBm\right)}\left(n-1\right)-X_{dB}, & \infty >P_{CS,k}^{\left(dBm\right)}\left(n-1\right)>P_{CS,LB}^{\left(dBm\right)}\\ P_{CS,UB}^{\left(dBm\right)}, & P_{CS,k}^{\left(dBm\right)}\left(n-1\right)=\infty \\ P_{CS,LB}^{\left(dBm\right)}, & P_{CS,k}^{\left(dBm\right)}\left(n-1\right)\leq P_{CS,LB}^{\left(dBm\right)} \end{array}\right.,\;k=1,\cdots K \end{array}$$

where $P_{CS,LB}^{\left(dBm\right)}$ is the lower bound of the variable range of the carrier sense level in the proposed method. Note that the second line of the right-hand term in Equation (9) indicates that the end devices that have not previously executed the carrier sense will do so.

These are summarized in Algorithm 1. In Algorithm 1, the proposed self-tuning algorithm is started after the ACK reception results have been accumulated for the memory length of $L_{M}$. Furthermore, the initial value of the carrier sense level is *∞*, that is, all the end devices do not execute the energy detection-based carrier sense in the initial state. This means that each end device will execute the carrier sense if necessary.
**Algorithm 1** Self-tuning algorithm at *k*th end device1:n←12:end of $L_{M}$ ACK results accumulation, i.e., $n\leftarrow L_{M}$3:$P_{CS,k}^{\left(dBm\right)}\left(L_{M}\right)\leftarrow \infty$4:**for all** $n\leftarrow L_{M}+1$ to $L_{M}+L_{P}$ **do**5: end of *n*th packet transmission6: calculate ${\hat{P}}_{PDR,k}\left(n\right)$ using Equation (7)7: **if** ${\hat{P}}_{PDR,k}\left(n\right)\geq \bar{P_{PDR}}$ **then**8:    calculate $P_{CS,k}^{\left(dBm\right)}\left(n\right)$ using Equation (8)9:  **else if** ${\hat{P}}_{PDR,k}\left(n\right)<\bar{P_{PDR}}$ **then**10:  calculate $P_{CS,k}^{\left(dBm\right)}\left(n\right)$ using Equation (9)11: **end if**12:**end for**

It is important to determine $P_{CS,LB}^{\left(dBm\right)}$ for efficient energy consumption at the end device. The energy detection-based carrier sense has an optimal carrier sense level where the best communication quality is obtained, and its characteristic has one peak at the optimal carrier sense level (the characteristics described below). Lowering the carrier sense level extends the carrier sense period, resulting in a carrier sense level below the optimal level of waste power of the end device. We let $P_{CS,OPT}^{\left(dBm\right)}$ denote the optimal carrier sense level in the network and define $P_{CS,LB}^{\left(dBm\right)}$ as $P_{CS,OPT}^{\left(dBm\right)}$.

The performances of the end devices with high path loss between the gateway and end devices, that is, poor conditioned end devices, are inferior to those of the rich conditioned end devices owing to the capture effect. In [29], the performance of the poor conditioned end devices can be improved by executing the energy detection-based carrier sense at the poor end devices in LPWANs because it can reduce the number of interferences for the end device executing the carrier sense. In the proposed self-tuning method, the carrier sense level of the end devices with high path loss to the gateway, that is, poor conditioned end devices, may be lower, whereas the carrier sense level of the end devices with low path loss to the gateway, that is, rich conditioned end devices, may be higher. Thus, the proposed method lowers the carrier sense level of the poor conditioned end devices to improve the performance.

## 5. Numerical Examples

### 5.1. Parameter Setup

**Figure 2:**
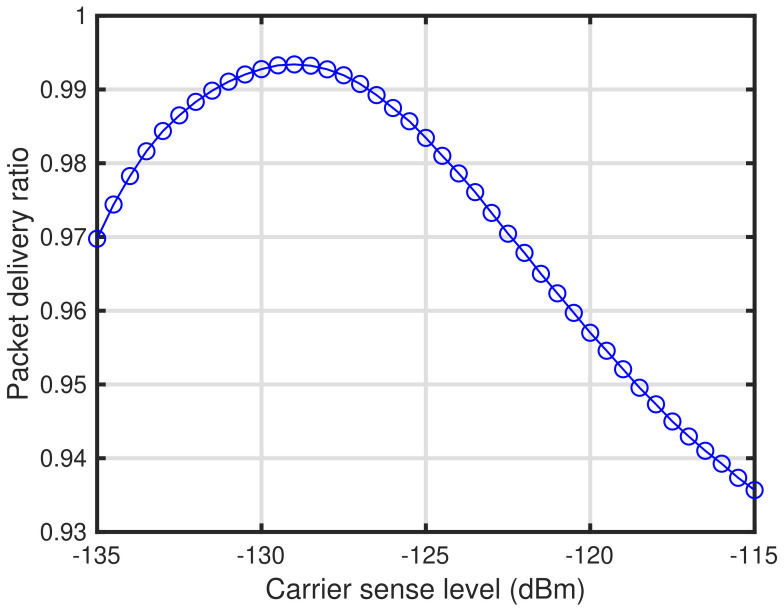
Characteristics of carrier sense levels versus packet delivery ratio.

**Figure 3:**
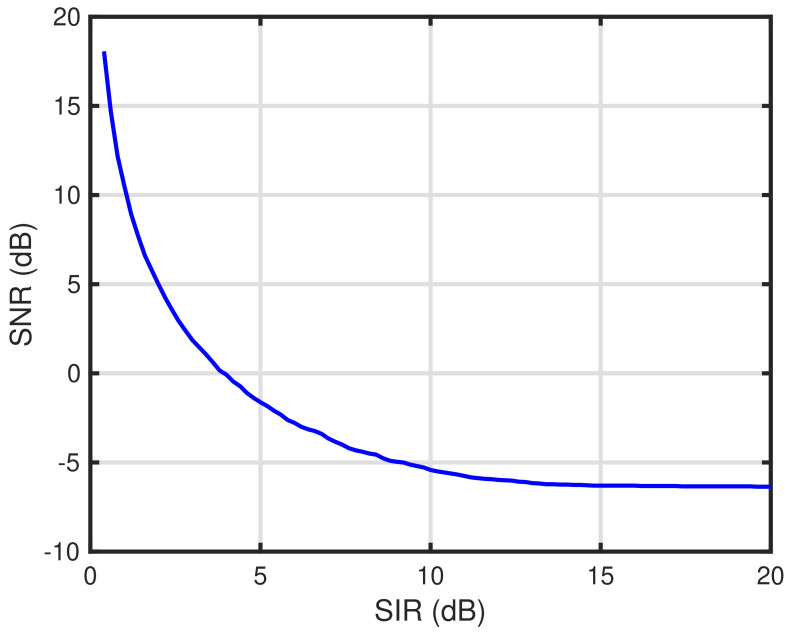
SIR-SNR relationship which determines the packet transmission success.

In this section, we show numerical examples to validate the effectiveness of the proposed self-tuning method. Table 2 lists the parameters of the numerical examples shown in this section. Numerical examples are obtained through computer simulations using MATLAB. In these simulations, K=200 end devices are deployed in the communication area with a radius R=1500 m. Assuming a suburban environment, we employ the path loss exponents between the end devices and gateway and between each end device as $\alpha_{GE}=2.7$ and $\alpha_{EE}=3.3$, respectively [30]. Note that these $\alpha_{GE}$ and $\alpha_{EE}$ are obtained as measurement results of radiowave propagation for smart metering in the suburban 950 MHz band, which is used before the 920 MHz band in Japan. We employ LoRa signals as the LPWA signals. Each end device transmits data via LoRa packets with a transmit power, length of packet, and spreading factor of 13 dBm, 153.9 ms, and 7, respectively. Note that the transmit power 13 dBm is defined as the maximum transmit power in the Japanese 920 MHz band [11] and the length of the LoRa packets follows the standard of LoRa Alliance [19], which is equivalent to conveying a payload of 50 bytes. The carrier frequency, noise figure at the receiver, and number of channels used are 920 MHz, 6 dB, and 1, respectively. Figure 2 shows the characteristics of the LPWAN with the energy detection-based carrier sense versus the packet delivery ratio based on the parameters listed in Table 2. As shown in Figure 2, it can be seen that the optimal carrier sense level that maximizes the packet delivery ratio is approximately −129 dBm. Therefore, we employ the lower bound of the variable range of the carrier sense level $P_{CS,LB}^{\left(dBm\right)}=-129$ dBm for the numerical examples shown in this section. Note that the details of the characteristics shown in Figure 2 are described in [12]. Furthermore, the upper bound of the variable range of the carrier sense level $P_{CS,UB}^{\left(dBm\right)}$ is −110 dBm. For the self-tuning, each end device equips memory for ACK results with length 128. The self-tuning is executed during the reception of 256 ACK packets at the end device. During the self-tuning period, the gateway transmits ACK via LoRa packets with a transmit power, length of ACK, and spreading factor of 13 dBm, 51.5 ms, and 7, respectively. Note that the length of the LoRa packets also follows the standard of LoRa Alliance [19], which is equivalent to conveying a payload of 1 bytes. The carrier sense level of the gateway is −129 dBm. For the signal detection parameters, the target signal detection probability and the target false alarm probability are 0.99 and 0.01, respectively. We employ the current consumption model for each operation of end devices as listed in Table 1. Figure 3 shows the SIR–SNR relationship which determines the packet transmission success. In Figure 3, the upper-region delimited by a curve represents the packet transmission success, whereas the lower-region delimited by a curve represents the packet transmission failure. The relationship is employed to determine whether the packet transmission is successful in the computer simulation.

### 5.2. Fundamental Characteristics of Self-Tuning Method

**Figure 4:**
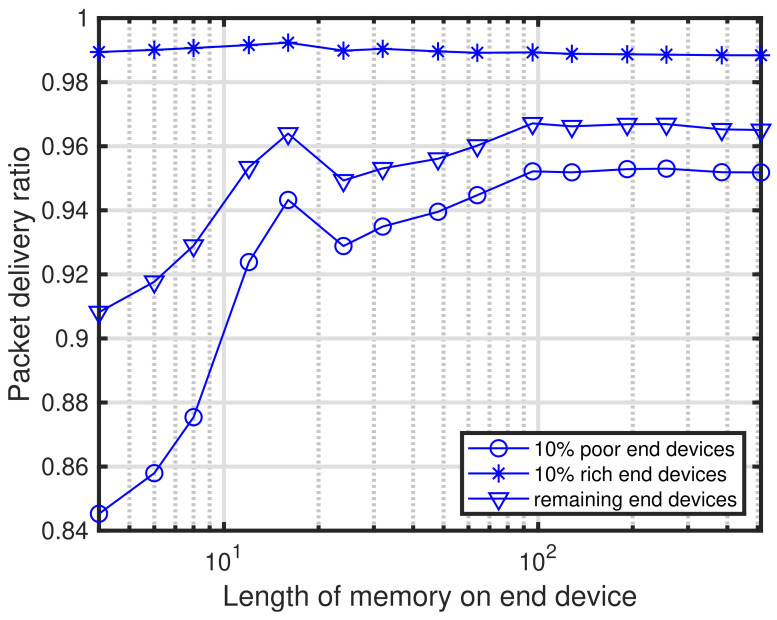
Length of memory for ACK results versus packet delivery ratio. $\bar{P_{PDR}}=0.95$.

**Figure 5:**
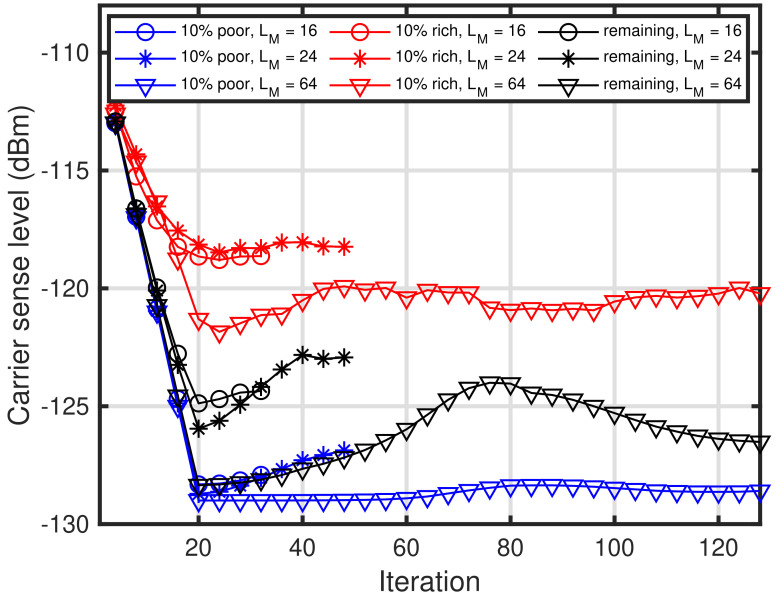
Convergence of carrier sense level. $L_{M}=16$, 24, 64 and $\bar{P_{PDR}}=0.95$.

In this subsection, several fundamental characteristics of the proposed self-tuning method are presented. First, we present the characteristics of the length of memory for ACK results $L_{M}$. Figure 4 shows the characteristics of the length of memory for ACK results versus the packet delivery ratio under $\bar{P_{PDR}}=0.95$. In Figure 4, three curves are depicted: 10% of the poor conditioned end devices, 10% of the rich conditioned end devices, and the remaining end devices. As shown in Figure 4, packet delivery ratios deteriorate when the length of memory exceeds 19. The reason for this is that it is insufficient a convergence for the self-tuning at the end devices, especially the 10% rich end devices. This is also shown in Figure 5. Furthermore, it can be seen that $L_{M}$ requires a certain length that is greater than 100.

**Figure 6:**
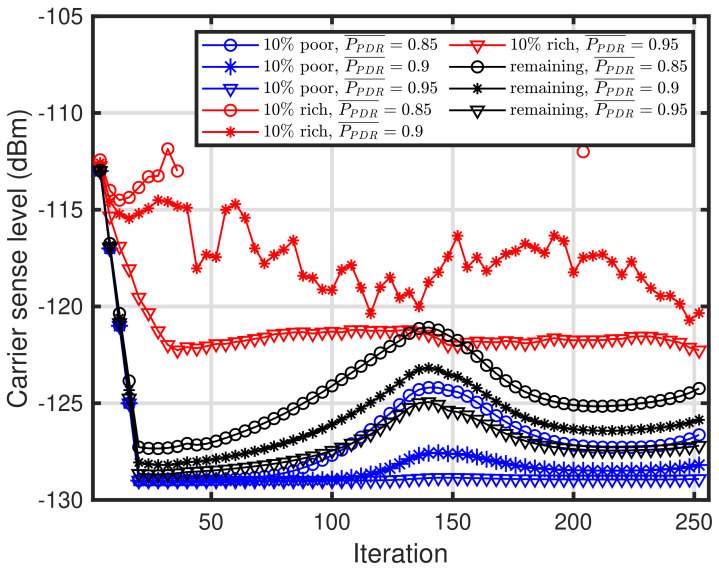
Convergence of carrier sense level. $\bar{P_{PDR}}=0.85$, 0.9, 0.95 and $L_{M}=128$.

Next, we present the characteristics of carrier sense level convergence using the proposed method. Figure 5 shows the convergence characteristics of the carrier sense level during the self-tuning periods. In Figure 5, the three types of curves are depicted as in Figure 4, and these curves correspond to $L_{M}=16$, 24, 64. Note that the self-tuning period, that is, the required number of ACKs, is twice as large as $L_{M}$. It can be seen that the carrier sense levels have not converged for $L_{M}=16$ and 24, whereas the carrier sense levels have converged for $L_{M}=64$. Furthermore, it can be seen that Figure 5 can explain Figure 4. Figure 6 shows the convergence characteristics of the carrier sense levels during the self-tuning periods for $L_{M}=128$. In Figure 6, the three types of curves are depicted as in Figure 4, and these curves correspond to $\bar{P_{PDR}}=0.85$, 0.9, and 0.95. As shown in Figure 4, the carrier sense levels for the end devices converge, except for the 10% of end devices in the rich environments. This is because almost all end devices in the rich environments do not require the carrier sense owing to the capture effect. Furthermore, the curve of the 10% rich condition for $\bar{P_{PDR}}=0.85$ represents that the energy detection-based carrier sense at the end devices is deemed unnecessary as soon as the iteration is less than 30.

**Figure 7:**
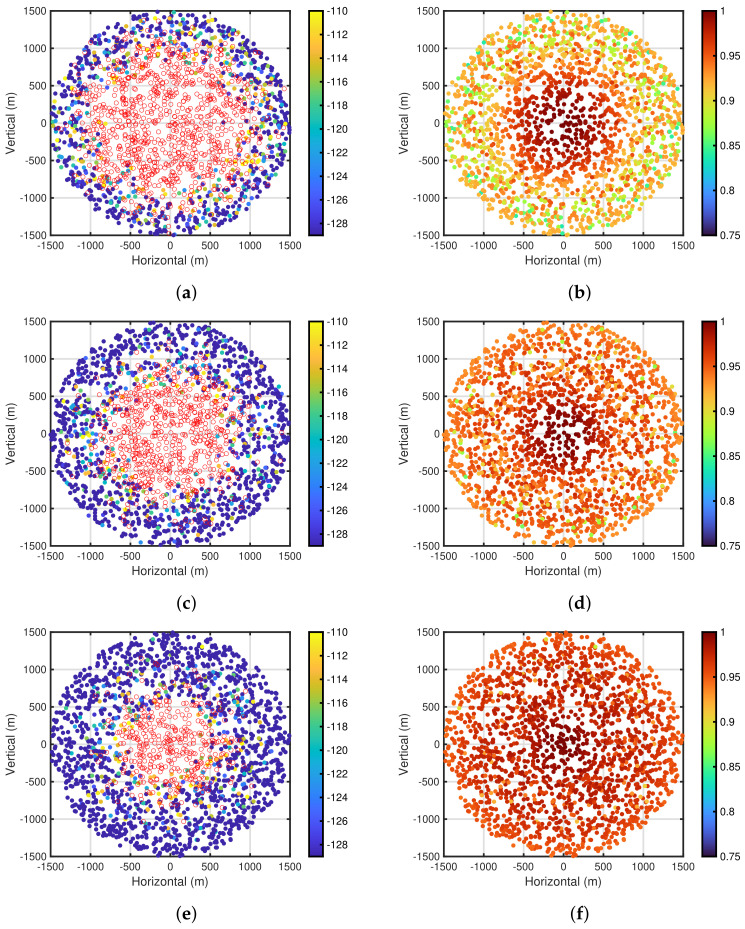
Carrier sense levels and packet delivery ratio for each end device. $N_{ED}=200$ and R=1500 m. (**a**) Carrier sense levels. $\bar{P_{PDR}}=0.85$; (**b**) Packet delivery ratios. $\bar{P_{PDR}}=0.85$; (**c**) Carrier sense levels. $\bar{P_{PDR}}=0.9$; (**d**) Packet delivery ratios. $\bar{P_{PDR}}=0.9$; (**e**) Carrier sense levels. $\bar{P_{PDR}}=0.95$; (**f**) Packet delivery ratios. $\bar{P_{PDR}}=0.95$.

To clarify these findings, Figure 7a,b show the carrier sense levels after the self-tuning period and packet delivery ratio based on the carrier sense levels for $\bar{P_{PDR}}=0.85$, $N_{ED}=200$, and R=1500 m, respectively. In both figures, the carrier sense levels and packet delivery ratio are depicted in the end device placement using color maps, respectively. Note that these Figure 7a,b are obtained by 10 computer simulation trials, and the end devices with no carrier sense are depicted by white circles with red lines in Figure 7a. As shown in Figure 7a,b, it can be seen that the performances of the centrally located end devices near the gateway are superior to other end devices, especially for the end devices near the outer perimeter of the communication area. However, the centrally located end devices do not execute the carrier sense. This indicates that the end devices in the rich environment do not execute the carrier sense by employing the proposed self-tuning method, which leads to the efficient energy consumption of such end devices. For the end devices with the carrier sense, it can be seen that the carrier sense levels of almost all end devices are the same at $P_{CS,LB}^{\left(dBm\right)}=-129$ dBm. Figure 7c,d show the carrier sense levels and packet delivery ratios for $\bar{P_{PDR}}=0.9$, respectively. It can be seen that the number of end devices with no carrier sense shown in Figure 7c is less than that shown in Figure 7a, and in conjunction with this finding, the performances of the packet delivery ratio in Figure 7d are superior to those shown in Figure 7b. This is because the poor conditioned end devices are included in the carrier sense range of other end devices, thereby reducing the number of interferences for the poor conditioned end devices [29]. Furthermore, Figure 7e,f show the performances for $\bar{P_{PDR}}=0.95$. A tendency similar to that shown in Figure 7a–d can be seen.

**Figure 8:**
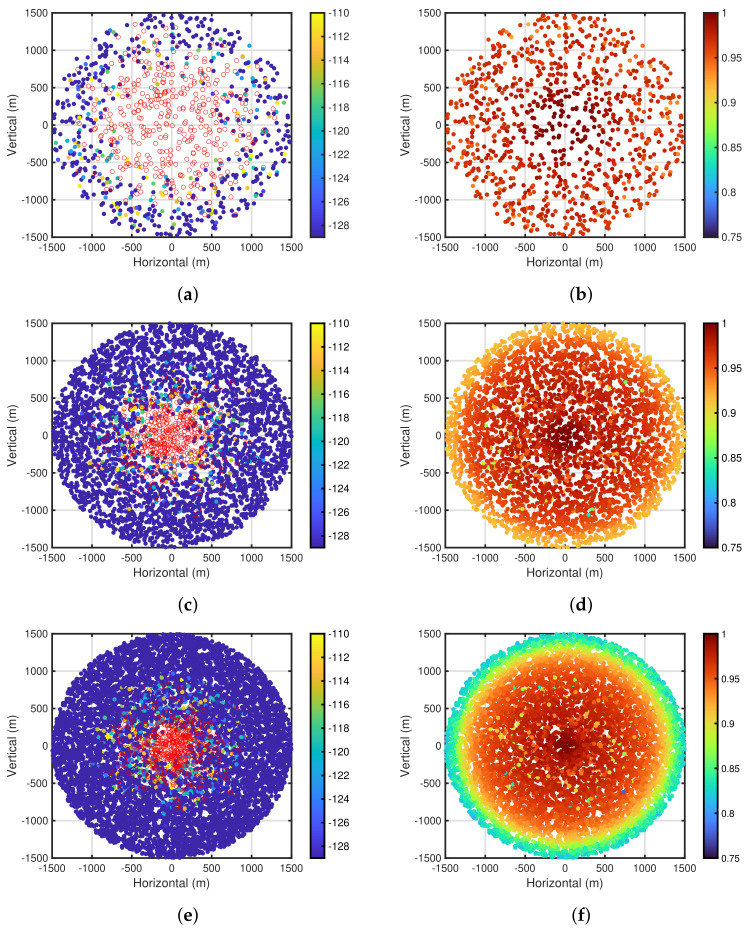
Carrier sense levels and packet delivery ratio for each end device. $\bar{P_{PDR}}=0.95$ and R=1500 m. (**a**) Carrier sense levels. $N_{ED}=100$; (**b**) Packet delivery ratios. $N_{ED}=100$; (**c**) Carrier sense levels. $N_{ED}=400$; (**d**) Packet delivery ratios. $N_{ED}=400$; (**e**) Carrier sense levels. $N_{ED}=800$; (**f**) Packet delivery ratios. $N_{ED}=800$.

Next, Figure 8a,b show the carrier sense levels after the self-tuning period and packet delivery ratio based on the carrier sense levels for $\bar{P_{PDR}}=0.95$, $N_{ED}=100$, and R=1500 m, respectively. In both figures, similar to Figure 7a–f, the carrier sense levels and packet delivery ratio are depicted in the end device placement using color maps, respectively. As shown in Figure 8a, the area, where the end devices that execute the carrier sense are present, is smaller than that shown in Figure 7e. This is because the decrease in $N_{ED}$ leads to a decrease in the number of interferences, resulting in a decrease in the number of the end devices that execute the carrier sense. On the other hand, Figure 8c–f show the carrier sense levels after the self-tuning period and packet delivery ratio for $\bar{P_{PDR}}=0.95$ and $N_{ED}=400$, 800, respectively. As shown in Figure 8c,e, the area, where the end devices that execute the carrier sense are present, is larger than that shown in Figure 7e, owing to the increase in the number of interferences. Furthermore, as shown in Figure 7f and Figure 8b,d,f, the packet delivery ratio of the poor conditioned end devices is deteriorated as $N_{ED}$ increases.

**Figure 9:**
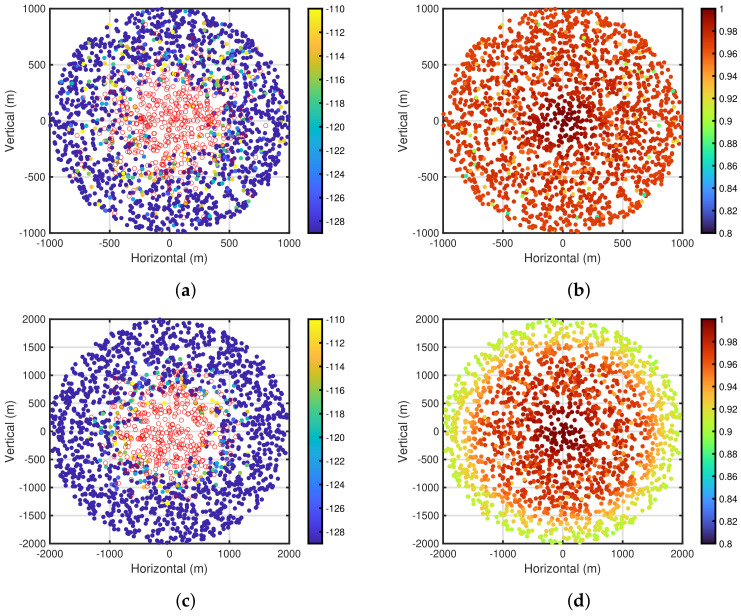
Carrier sense levels and packet delivery ratio for each end device. $\bar{P_{PDR}}=0.95$ and $N_{ED}=200$. (**a**) Carrier sense levels. R=1000 m; (**b**) Packet delivery ratios. R=1000 m; (**c**) Carrier sense levels. R=2000 m; (**d**) Packet delivery ratios. R=2000 m.

Finally, Figure 9a–d show the carrier sense levels after the self-tuning period and packet delivery ratio based on the carrier sense levels for R=1000 m and R=2000 m, respectively. Note that the results are obtained under $\bar{P_{PDR}}=0.95$ and $N_{ED}=200$. As shown in Figure 7e and Figure 9a,c, the areas, where the end devices that execute the carrier sense are present, in the three figures are almost the same. This is because the relationship itself between the received signal power from the end devices does not depend on the radius of the communication area. On the other hand, as shown in Figure 7f and Figure 9b,d, the performances of the packet delivery ratio are deteriorated as the radius *R* increases. This is because the number of end devices outside the carrier sense range, that is, hidden terminals, increases as the radius *R* increases.

### 5.3. Characteristics of Proposed Self-Tuning Method for Channel Changing Owing to Some Factors

In this subsection, we show the evaluation results of the proposed self-tuning method for channel characteristics that have changed owing to some factor. We let $\sigma_{C}$ denote a standard deviation of channel changing. Using $\sigma_{C}$, the changed path loss $\tilde{L_{k}}$ between the gateway and *k*th end device in decibels is defined as follows.

(10) $$\begin{array}{c} \tilde{L_{k}}=L_{k}+x,\;k=1,\cdots ,K, \end{array}$$

where *x* is a random variable which follows Gaussian distribution with zero mean and standard deviation $\sigma_{C}$ in decibels. Similarly, the changed path loss $\tilde{\Theta_{k,i}}$ between *k*th and *i*th end devices in decibels can be defined as follows:

(11) $$\begin{array}{c} \tilde{\Theta_{k,i}}=\Theta_{k,i}+x,\;k\neq i,\;k,i=1,\cdots ,K. \end{array}$$

**Figure 10:**
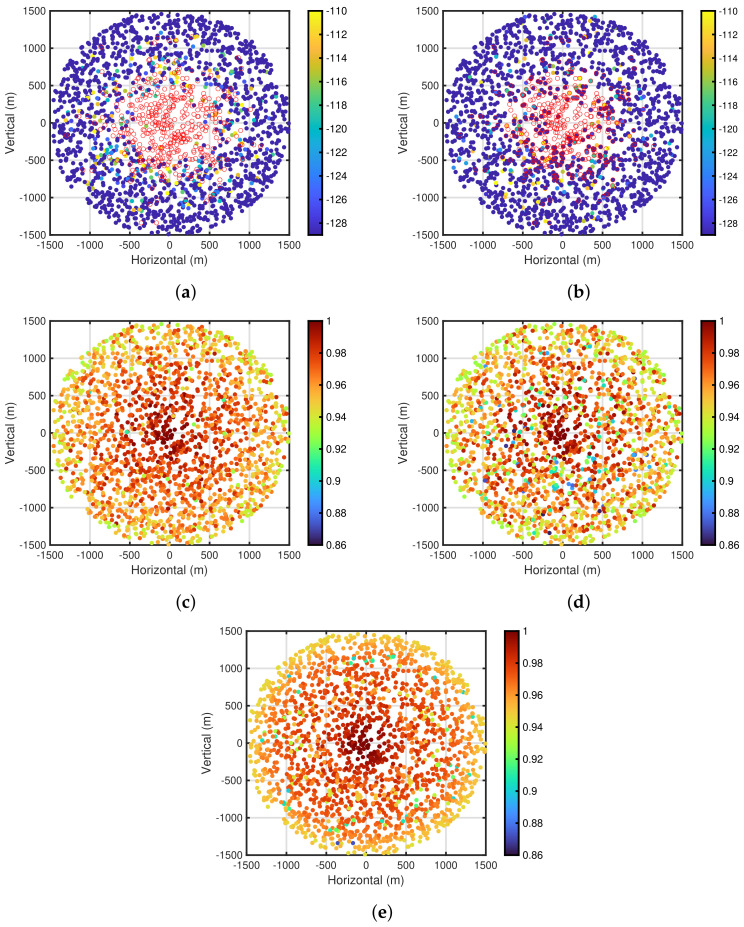
Carrier sense levels and packet delivery ratios for each end device. $\bar{P_{PDR}}=0.95$, $N_{ED}=200$, R=1500 m, and $\sigma_{C}=7.5$ dB. (**a**) Carrier sense levels before changing; (**b**) Carrier sense levels after changing; (**c**) Packet delivery ratios with tracking; (**d**) Packet delivery ratios without tracking; (**e**) Packet delivery ratios before changing.

Using $\tilde{L_{k}}$ and $\tilde{\Theta_{k,i}}$, the characteristics of the proposed self-tuning method for the channel changing are evaluated. The evaluations are carried out under $\bar{P_{PDR}}=0.95$, $N_{ED}=200$, R=1500 m, and $\sigma_{C}=7.5$ dB. Figure 10a,b show the carrier sense levels before channel changing and after channel changing, respectively. Note that the carrier sense levels in Figure 10a,b are obtained by the proposed method for the channel before and after changing, respectively. As shown in both figures, the carrier sense levels in Figure 10b are changed compared to those in Figure 10a. Figure 10c,d show the packet delivery ratios. Figure 10c shows the packet delivery ratios when tracking the channel changing, whereas Figure 10d shows those without tracking the channel changing. It can be seen that the characteristics in Figure 10c are superior to those in Figure 10d. Furthermore, Figure 10e shows the packet delivery ratio for the channel before changing with the carrier sense levels shown in Figure 10a. Note that, although Figure 7f and Figure 10e are fundamentally the same, the values in both figures allocated to the color map are only different for the comparison. As shown in Figure 10c,e, both characteristics are almost the same. This indicates that the proposed self-tuning method can track the channel changing by periodically tuning the carrier sense level.

### 5.4. Characteristics of Self-Tuning Method Regarding Energy Consumption

**Figure 11:**
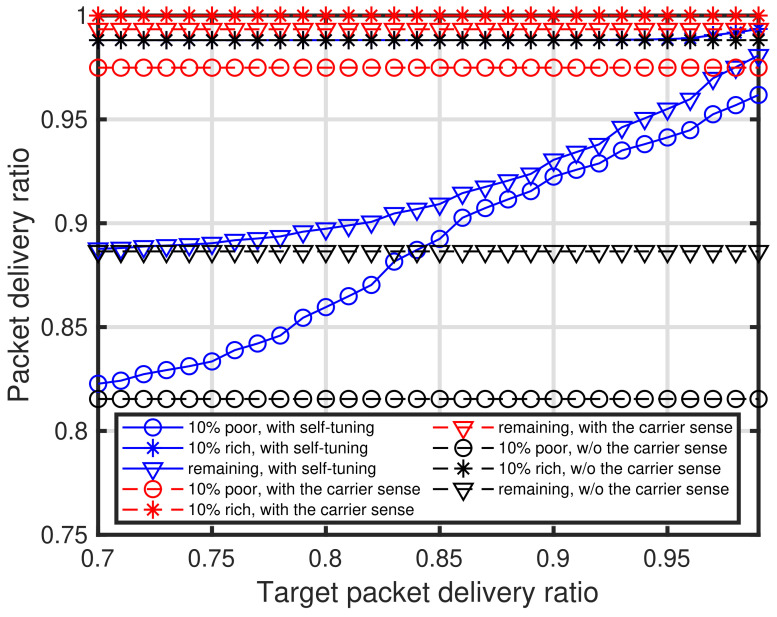
Characteristics of target packet delivery ratio versus packet delivery ratio.

**Figure 12:**
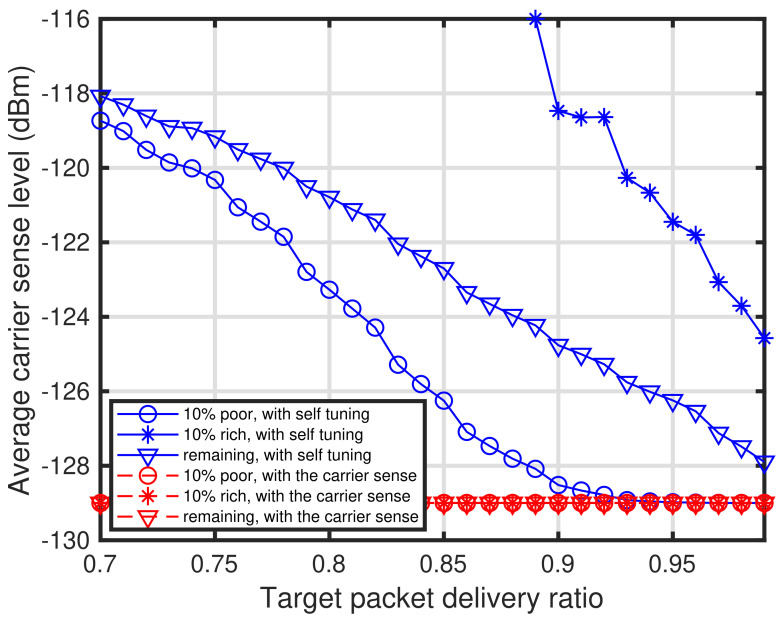
Characteristics of target packet delivery ratio versus average carrier sense level.

Figure 11 shows the characteristics of the target packet delivery ratio $\bar{P_{PDR}}$ versus the packet delivery ratio. In Figure 11, the characteristics of the three types of end devices in the three types of networks, that is, the networks with self-tuning, with the energy detection-based carrier sense, and without any carrier sense method. For the characteristics of the network with the energy detection-based carrier sense, the carrier sense level of all the end devices is −129 dBm. As shown in Figure 11, the performance of the end devices with the proposed self-tuning approaches those obtained with the carrier sense. Furthermore, Figure 12 shows the characteristics of the target packet delivery ratio versus the average carrier sense level. It can be seen that the carrier sense level decreases as the target packet delivery ratio increases. Furthermore, it can be seen that the rich conditioned end devices do not execute the carrier sense when $\bar{P_{PDR}}<0.89$.

**Figure 13:**
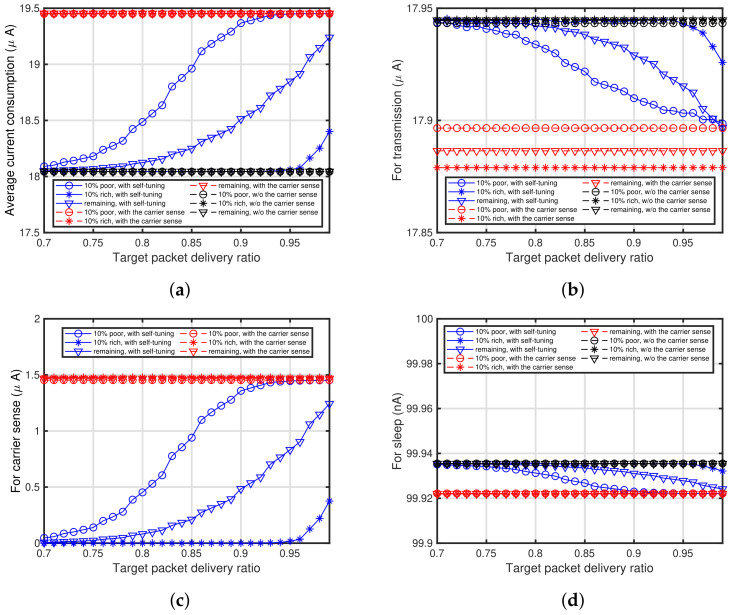
Characteristics of target packet delivery ratio versus average current consumption for each operation. (**a**) All operations; (**b**) Packet transmission; (**c**) Carrier sense; (**d**) Sleep.

**Figure 14:**
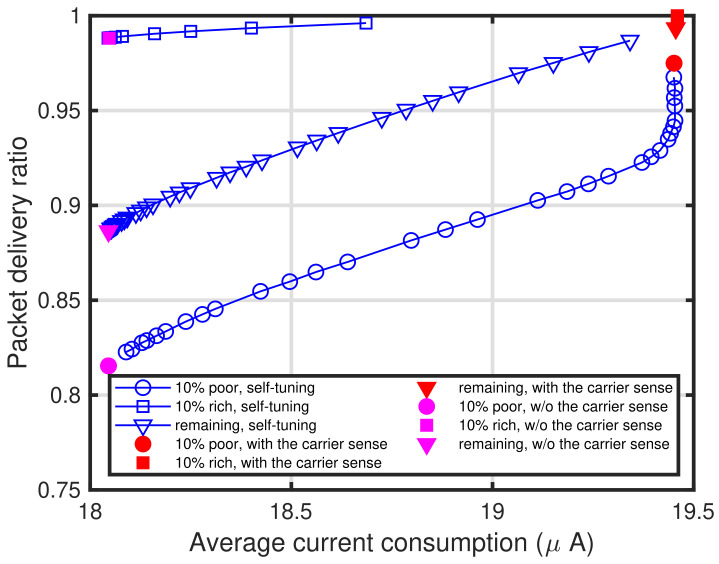
Trade-off in proposed self-tuning method between average current consumption and packet delivery ratio for different $\bar{P_{PDR}}$s.

Figure 13a shows the characteristics of the target packet delivery ratio versus the average current consumption. Note that the analytical results for current consumption shown in this subsection are based on Table 1. It can be seen the current consumption of 10% for the poor conditioned end devices using the proposed self-tuning method is almost the same as that of the end devices with the carrier sense for a high target packet delivery ratio. However, the consumption of the remaining 90% of end devices, especially the 10% rich conditioned end devices, is less than that of the end devices with the carrier sense. To show the details of the current consumption, Figure 13b–d show the characteristics of the average current consumption for packet transmission, carrier sense, and sleep, respectively. Note that the legends of Figure 13b–d are identical to those in Figure 13a. As shown in Figure 13c, the average current consumption of the end devices with the proposed method is less than that of the end devices with the energy detection-based carrier sense, whereas the average current consumption of the end devices with the proposed method in Figure 13b,d is almost the same as that of the end devices with the energy detection-based carrier sense. Especially, it can be seen that the current consumption of the poor conditioned end devices is greater than the other end devices, whereas the consumption of the rich conditioned end devices is less than the other end devices. Hence, the end devices’ performances to be improved, that is, the poor conditioned end devices, require a large amount of energy when adopting the proposed self-tuning method. Furthermore, the proposed self-tuning method does not consume much energy of the end devices whose performance does not need to be improved, that is, the rich conditioned end devices. To show these clearly, Figure 14 shows the trade-off in the proposed self-tuning method between the average current consumption and packet delivery ratio. In Figure 14, the relationship between the average current consumption and packet delivery ratio are depicted for different $\bar{P_{PDR}}$s. It can be seen that these are rather straightforward and obvious.

## 6. Conclusions

In this study, we proposed the self-tuning method of the signal detection level of the energy detection-based carrier sense. The proposed self-tuning method enables the autonomous decentralized determination of the carrier sense level at each end device. To achieve this, the proposed method was utilized whether ACK packet transmissions were successful or not. The proposed self-tuning method does not require the development of new protocols. The numerical examples demonstrated that the proposed self-tuning method can improve the performance of the end devices whose performance is to be improved. Furthermore, the proposed self-tuning method leads to rather straightforward and obvious results according to which the end devices whose performance does not need to be improved do not consume much energy, that is, the rich conditioned end devices by employing the proposed self-tuning method.

The future work involves the investigation of a self-tuning method of transmit power at each end device and the development of a hybrid self-tuning method of transmit power and signal detection level of the energy detection-based carrier sense.

## Figures and Tables

**Table 1:** Current consumption of end devices for each operation.

Parameter	Value
Packet transmission	35 mA
Carrier sense	10.8 mA
Sleep	100 nA

**Table 2:** Parameters for numerical examples of LPWANs.

Parameter	Variable	Value
Number of end devices	$N_{ED}$	200
Radius of communication areas	*R*	1500 m
Path loss exponent	$\alpha_{GE}$	2.7 (suburban)
(end device–gateway)		
Path loss exponent	$\alpha_{EE}$	3.3 (suburban)
(end device–end device)		
Transmit power at end device	$P_{TX}$	13 dBm
Average transmission period	$T_{TX}$	300 s
(Poisson distribution)		
Length of packet		153.9 ms
Spreading factor (end devices)		7
Maximum carrier sense time		3
Carrier frequency	$f_{c}$	920 MHz
Noise figure at receiver	NF	6 dB
Number of channels used	−	1
Lower bound of carrier sense level	$P_{CS,LB}^{\left(dBm\right)}$	−129 dBm
Upper bound of carrier sense level	$P_{CS,UB}^{\left(dBm\right)}$	−110 dBm
Length of memory for ACK result	$L_{M}$	128
Length of self-tuning period	$L_{P}$	256 ACKs
Transmit power at gateway	−	13 dBm
Length of ACK	−	51.5 ms
Spreading factor (gateway)		7
Carrier sense level at gateway	−	−129 dBm
Target signal detection probability	$\bar{P_{D}}$	0.99
Target false alarm probability	$\bar{P_{FA}}$	0.01
Current consumption of end devices	−	Table 1
Capture effect	−	Figure 3

## Data Availability

The original contributions presented in the study are included in the article, further inquiries can be directed to the corresponding author.
